# Blood pressure and electrocardiographic profile of children with sickle cell anaemia in steady-state and vaso-occlusive crisis

**DOI:** 10.4314/gmj.v57i3.4

**Published:** 2023-09

**Authors:** Adewuyi T Adeniyi, Samuel A Adegoke, Oladele S Olatunya, Adefunke O Babatola, Adebukola B Ajite, Ezra O Ogundare, Isaac O Oluwayemi, Adedolapo A Abe-Dada, John A O Okeniyi

**Affiliations:** 1 Department of Paediatrics and Child Health, Ekiti State University, Ado-Ekiti, Nigeria; 2 Department of Paediatrics and Child Health, Ekiti State University Teaching Hospital, Ado-Ekiti, Nigeria; 3 Department of Paediatrics and Child Health, Obafemi Awolowo University, Ile-Ife, Nigeria; 4 Department of Paediatrics and Child Health, Obafemi Awolowo University Teaching Hospitals Complex, Ile-Ife, Nigeria

**Keywords:** blood pressure, electrocardiographic, sickle cell anaemia, vaso-occlusive crisis

## Abstract

**Objective:**

To compare the cardiovascular features of patients with sickle cell anaemia (SCA) in steady-state with those in vaso-occlusive crisis (VOC) at the Wesley Guild Hospital (WGH).

**Design:**

A descriptive cross-sectional, matched, case-control study among children with SCA at the WGH, a tertiary health facility in southwest Nigeria.

**Setting:**

The participants were recruited from the children's emergency unit and paediatric haematology clinic of the WGH.

**Participants:**

Consisted of 93 children with VOC (cases) and 93 age and sex-matched in steady state (controls), aged 5 – 15 years.

**Main outcome measures:**

Cardiovascular parameters, including pulse rate, blood pressure, and electrocardiographic profile, were assessed and compared using the appropriate statistical tests.

**Results:**

The mean (SD) age of the cases and controls were 8.8 (3.2) years and 9.0 (3.1) years, respectively (p= 0.106). There was no significant difference in the mean height of the groups. The mean pulse rate, diastolic, systolic, and mean arterial pressures were significantly higher in the cases than in the controls. A significantly higher proportion of the cases than the controls also had a higher frequency of heart blocks, prolonged QTc interval, ST elevation or depression, and T wave abnormality (p = 0.018, 0.039, 0.041, 0.009, respectively). The prevalence of chamber enlargements was not significantly different between the two groups.

**Conclusion:**

Cardiovascular dysfunction is worse during VOC when compared with steady state. Physicians should look for these dysfunctions in SCA children with VOC to reduce mortality from the disease.

**Funding:**

None declared

## Introduction

Sickle cell anaemia (SCA) is a chronic illness most prevalent in sub-Saharan Africa.^1^ About 3.4 million Nigerians are reported to have sickle cell anaemia, and a prevalence rate of 1% was reported in children.^2^ Notwithstanding the efforts to reduce the disease's prevalence and mortality,^1^,^3^ SCA remains a significant cause of morbidity and mortality in sub-Saharan Africa, Southeast Asia and India.^4^,^5^

The pathophysiologic mechanism in SCA involves recurrent vaso-occlusion by sickled, poorly pliable red blood cells (RBC), recurrent haemolysis and shortened RBC life span, vascular endothelial changes, and depletion of intravascular nitric oxide, among others.^6^,^7^ These pathophysiologic aberrations affect all vascular beds and result in long-term complications in all organ systems. Recent studies of the cardiovascular system in SCA have suggested hyperdynamic and superimposed restrictive physiology.^8^–^10^ The roles of increased oxidative stress have also been described.^11^

Cardiovascular complications have been widely reported in SCA patients in steady-state. These include chamber dilatations and hypertrophy, arrhythmias, myocardial ischaemia, electrocardiographic (ECG) abnormalities, diastolic dysfunction and pulmonary hypertension.^12^–^16^ Blood pressure in SCA patients in a steady-state is usually lower than in haemoglobin AA phenotype.^17^,^18^ Transient blood pressure increase may, however, occur during a vaso-occlusive crisis (VOC), and this has been implicated in the occurrence of stroke in SCA.^19^

Most studies on cardiovascular changes in SCA have been conducted in patients in steady state, compared with apparently healthy haemoglobin AA patients. Few studies have, however, shown that these abnormalities are worse during VOC and may further complicate this acute clinical state in such patients, thus increasing the risk of mortality.^18^,^20^ The treatment of SCA patients in VOC often focuses on adequate hydration and pain management and neglects the assessment of complications in other organs.^21^ This study set out to describe the cardiovascular findings during VOC in comparison with steady state. The null hypothesis is that there are no significant differences in the VOC and steady-state cardiovascular findings.

## Methods

### Study design and study population

The study was a descriptive, cross-sectional, matched case-control study conducted between February and November 2017 at the Wesley Guild Hospital (WGH), a unit of the Obafemi Awolowo University Teaching Hospital Complex (OAUTHC), southwest Nigeria. The WGH is in Ilesa and provides tertiary health care to paediatric patients and adults.

The minimum number of participants required for the study was calculated using the formula for determining proportions in a comparative study.^22^ A study by Bode-Thomas et al. in Nigeria determined the proportion (p) for the cases and controls applicable in the sample size formula.^23^ A total of 186 children with sickle cell anaemia (SCA) aged between 5 and 15 years were studied. These included 93 children (cases) who presented with vaso-occlusive crisis (VOC) and 93 age and sex-matched controls recruited in their steady states. VOC was defined as the presence of an “acute episode of pain requiring hospital visits and treatment with an analgesic”, while “steady-state” was defined as “a period during which the patient has no acute event such as pain, fever or infection in the last 4 weeks and no blood transfusion in the last 3 months”.^24^ The cases were recruited from the children's emergency unit of WGH, while the controls were recruited during their routine follow-up visits at the Paediatric Haematology clinic of the WGH.

For both study groups, children who had previously been diagnosed with congenital or acquired heart disease and those who had used medications known to produce ECG changes in the last 7 days before contact was excluded. Participants who had significant anaemia (mixed crisis) were excluded because significant anaemia could produce ECG changes related to ischaemia. Similarly, cases that presented more than 48 hours after the onset of VOC were also excluded since some cardiovascular and ECG changes of ischaemia could have begun to resolve by then. All the children recruited in VOC (cases) were excluded from being recruited as controls during their follow-up visits.

### Sampling procedure and data collection

Consecutive cases and controls who met the inclusion criteria were recruited. Each case was matched with a control of the same gender and whose age is within six months of the age of the case, except for three cases for whom we could not find a perfect match among our patients within the study period. Information about the age, gender, and year of diagnosis of sickle cell anaemia was obtained from each participant. For cases, the onset and duration of painful episodes and interventions given before presentation were obtained. A full cardiovascular examination of each participant was done. Each participant's height was measured using a stadiometer to the nearest 0.1 centimetres. The length was measured for participants with pain in the lower limbs who could not stand without support. The participant's blood pressure was measured on the right arm with an Accosson mercury sphygmomanometer, using an appropriate cuff, after at least 15 minutes of bed rest. Two blood pressure readings were done 5 minutes apart, and the two values' averages were recorded. Electrocardiography (ECG) was done with Biocare IE12A mobile ECG machine. ECG was done between 12 and 48 hours after the onset of VOC for cases while it was done at contact for controls. Participants rested for 15 minutes before the ECG recordings were made. ATA interpreted the ECG readings, while JAOO, a consultant paediatric cardiologist, did a second interpretation. The concordance between the two interpretations was above 95%. The ECG recordings were analysed by age groups (5 – 7, 8 – 10, 11 – 13 and 14 – 15 year-olds) to reflect known variations in ECG parameters with age. Values were also compared with standard values for the study environment as described by Kolawole and Omokhodion.^25^ To exclude significant differences in haematocrit as a confounding factor, the haematocrit of each participant (cases and controls) was determined from a venous blood sample. The ECG recordings were taken before the venous blood samples were obtained. Blood pressure values above the 90^th^ percentile on the 95^th^ percentile height value for age and sex were considered hypertensive.

In contrast, values below the 50^th^ percentile on the 5^th^ percentile height value for age and sex or an absolute systolic blood pressure below 50 mmHg were regarded as hypotensive.^26^

### Ethical considerations

Ethical clearance for the study was obtained from the Ethics and Research Committee of the OAUTHC (ERC/2016/06/10). A signed, written informed consent was obtained from all the caregivers of the participants. An assent for participation was obtained from those 7 years or older.

### Data management and statistical analysis

Data analysis was done with the Statistical Package for the Social Sciences version 20.0 (IBM Corporation 2011). Means, medians, standard deviations and interquartile ranges were calculated for continuous variables, while proportions and percentages were calculated for categorical variables. Comparisons between means were made with Student t-test and between proportions with Pearson chi-square.

## Results

Ninety-three cases with vaso-occlusive crisis and 93 age and sex-matched controls in steady state were recruited. The mean ages (SD) of the cases and controls were 8.8 (3.2) years and 9.0 (3.1) years (t = -1.623, p = 0.106). The cases consisted of 54 males and 39 females, while there were 51 male and 42 female controls. There was no statistically significant difference in the sex distribution between the two study groups (p = 0.657). For the cases, the mean duration of pain before presentation was 13.0 (5.0) hours. There was no significant difference in the haematocrit between the two groups. The haematocrit of the cases ranged from 18 to 37%, while it ranged from 17 to 36% for the controls [mean 24.3 (5.5) % versus 24.8 (4.2) %, t = - 0.589, p = 0.557]. There was no significant difference in the mean (SD) height of the cases, 125.0 (15.4) cm and the controls, 127.9 (14.9) cm, t = -1.327, p = 0.186. The mean radial pulse rate, systolic, diastolic, and mean arterial blood pressures were significantly higher in the cases than in the controls, p = < 0.001, 0.012, < 0.001 and < 0.001, respectively (Table 1).

**Table 1:** Comparison of the heart rate and blood pressure parameters between the cases and controls

Heart rate and blood pressure parameters	Children with VOC (93) Mean (SD)	Children in steady state (93) Mean (SD)	t-test	p-value
**a. Heart rate (bpm)**	104.8 (21.5)	94.4 (14.5)	3.854	**<0.001**
**b. Systolic Blood Pressure (mmHg)**	98.4 (10.6)	94.7 (9.0)	2.552	**0.012**
**c. Diastolic Blood Pressure (mmHg)**	60.0 (9.1)	54.9 (8.8)	3.933	**<0.001**
**d. Mean Arterial Pressure (mmHg)**	72.8 (9.15)	68.2 (7.57)	3.790	**<0.001**

bpm:- beats per minute; mmHg:- millimetres of mercury.

The mean ECG-determined heart rate (in beats per minute) gradually decreased with age in both groups. It was also higher in the cases than the controls in each age group but was significantly so only among the 5 – 7 year-olds (t = 4.714, p < 0.001). The mean corrected QT interval (QTc) was generally prolonged (≥ 0.44 seconds) in both cases and controls but not significantly different in any of the age groups. Other ECG parameters assessed included the mean P wave duration, PR interval, P axis, QRS axis, R wave voltage in Lead 5 and S wave voltage in Lead 1. All these parameters were not significantly different between the cases and the controls in all the age groups. Details of these are shown in Table 2. Most participants in both study groups had normal blood pressure (92.5% for cases and 96.8% for controls). Four (4.3%) cases had hypertension, and none in the control group. (Table 3).

**Table 2a:** Comparison of age-dependent mean electrocardiographic indices of cases and controls

ECG indices	5 – 7 year olds				8 – 10 year olds			
	VOC N = 40	Steady-state N = 38	t-test	p-value	VOC N = 28	Steady-state N = 27	t-test	p-value
**Heart rate (bpm)**	110.6 (15.3)	95.9 (9.9)	4.714	**0.000**	93.6(8.5)	89.7 (14.0)	1.097	0.278
**P-wave duration (sec)**	0.07 (0.01)	0.08 (0.01)	-0.697	0.488	0.08 (0.01)	0.07 (0.01)	2.167	0.053
**PR interval (sec)**	0.13 (0.02)	0.14 (0.02)	-1.159	0.250	0.15 (0.03)	0.14 (0.02)	1.914	0.061
**QT interval (sec)**	0.33 (0.03)	0.35 (0.02)	-2.891	0.136	0.38 (0.03)	0.36 (0.03)	1.023	0.311
**QTc interval (sec)**	0.45 (0.02)	0.44 (0.02)	0.663	0.509	0.46 (0.03)	0.44 (0.02)	2.736	0.080
**P axis (** ^**o**^ **)**	48.9 (24.3)	33.0 (24.2)	2.847	0.054	39.4 (23.6)	31.9 (22.3)	1.133	0.262
**QRS axis (** ^**o**^ **)**	47.5 (15.8)	50.4 (13.8)	-0.849	0.399	43.7 (28.0)	45.7 (24.7)	-0.275	0.784
**T axis (** ^**o**^ **)**	39.8 (16.1)	46.3 (15.2)	-1.810	0.074	39.1 (17.3)	39.3 (18.1)	-0.044	0.965
**RV5 voltage (mV)**	1.42 (0.50)	1.61 (0.50)	-1.714	0.091	2.78 (0.62)	2.83 (0.91)	-0.202	0.841
**SV1 voltage (mV)**	1.18 (0.65)	1.28 (0.62)	-0.680	0.498	1.06 (0.47)	1.01 (0.46)	0.364	0.717
**RV5 + SV1 voltage (mV)**	3.67 (1.03)	3.99 (0.91)	-1.384	0.170	3.85 (0.77)	3.84(1.04)	0.004	0.997

Values in parentheses represent percentages along each column.

**Table 2b:** Comparison of age-dependent mean electrocardiographic indices of cases and controls

ECG indices	11 – 13 year olds				14 - 15 year olds			
	VOC N = 15	Steady-state N = 17	t-test	p-value	VOC N = 10	Steady-state N = 11	t-test	p value
**Heart rate (bpm)**	93.1 (26.2)	87.8 (12.8)	0.766	0.449	84.2 (14.6)	81.4 (7.60)	0.539	0.597
**P-wave duration (sec)**	0.09 (0.02)	0.08 (0.01)	1.181	0.246	0.08 (0.02)	0.08 (0.01)	0.554	0.587
**PR interval (sec)**	0.15 (0.03)	0.16 (0.02)	-1.297	0.204	0.15 (0.02)	0.16 (0.02)	-0.673	0.510
**QT interval (sec)**	0.36 (0.04)	0.37 (0.02)	-0.073	0.943	0.38 (0.03)	0.37 (0.03)	0.510	0.616
**QTc interval (sec)**	0.45 (0.02)	0.44 (0.02)	0.654	0.518	0.45 (0.03)	0.44 (0.03)	1.383	0.184
**P axis (** ^**o**^ **)**	41.5 (20.9)	42.9 (18.1)	-0.208	0.837	48.8 (19.1)	45.7 (21.0)	0.345	0.734
**QRS axis (** ^**o**^ **)**	40.9 (11.5)	31.8 (25.5)	1.274	0.212	51.7 (11.5)	45.7 (23.0)	0.736	0.471
**T axis (** ^**o**^ **)**	30.1 (13.5)	32.4 (15.9)	-0.416	0.680	34.6 (17.3)	35.0 (40.3)	-0.029	0.977
**RV5 voltage (mV)**	2.85 (0.84)	2.65 (1.00)	0.602	0.551	3.08 (0.79)	2.94 (0.81)	0.374	0.713
**SV1 voltage (mV)**	1.61 (0.70)	1.56 (0.39)	2.341	0.626	1.11 (0.42)	1.05 (0.45)	0.335	0.741
**RV5 + SV1 voltage (mV)**	4.46 (1.19)	3.81 (1.09)	1.629	0.113	4.20 (0.96)	4.00 (0.91)	0.475	0.640

Values in parentheses represent percentages along each column.

**Table 3:** Prevalence of blood pressure abnormalities among the participants

Blood Pressure	Children with VOC (93) N (%)	Children in steady state (93) N (%)	x^2^	p-value
**a. Normal**	86 (92.5)	90 (96.8)		
**b. Hypertensive**	4 (4.3)	0 (0.0)	5.636*	0.060
**c. Hypotensive**	3 (3.2)	3 (3.2)		
**Total**	93 (100)	93 (100)		

* analysed with chi-square with likelihood ratio

As shown in Table 4, the most common ECG abnormality found in the participants was left ventricular hypertrophy (LVH). Among the cases, LVH was found in 45.0%, 57.1%, 66.7% and 40.0% of the 5 – 7 year-olds, 8 – 10 year-olds, 11 – 13 year-olds and 14 – 15 year-olds, respectively. For the controls, LVH was found in 60.5%, 63.0%, 50.0%, and 40.0% of the same age ranges, respectively.

**Table 4a:** Age-related prevalence of chamber and conduction abnormalities between the two groups

ECG abnormality	5 – 7 year-old			8 – 10 year-old		
	VOC N = 40	Steady-state N=38	p-value	VOC N = 28	Steady-state N=27	p-value
**LVH**	18 (45.0)	23 (60.5)	0.170	16 (57.1)	17 (63.0)	0.660
**RVH**	3 (7.5)	3 (7.9)	0.719^#^	4 (14.3)	2 (7.4)	0.149^#^
**BVH**	2 (5.0)	2 (5.3)	0.645^#^	3 (10.7)	2 (7.4)	0.966^#^
**RAE**	3 (7.5)	0 (0.0)	0.257^#^	0 (0.0)	1 (3.7)	0.985^#^
**LAE**	2(5.0)	0 (0.0)	0.497^#^	2 (7.1)	0 (0.0)	0.488^#^
**Axis deviation**						
**Absent**	40 (100.0)	38 (100.0)	-	24 (85.7)	24 (88.9)	0.723*
**LAD**	0 (0.0)	0 (0.0)		4 (14.3)	3 (11.1)	
**Heart blocks**						
**Absent**	36 (90.0)	36 (94.7)	0.428*	18 (64.3)	25 (92.6)	0.027*
**First degree**	4 (10.0)	2 (5.3)		7 (25.0)	1 (3.7)	
**Second degree**	0 (0.0)	0 (0.0)		3 (10.7)	1 (3.7)	
**Bundle branch blocks**						
**Absent**	35 (87.5)	35 (92.1)	0.500*	26 (92.9)	25 (92.6)	0.970*
**RBBB**	5 (12.5)	3 (7.9)		2 (7.1)	2 (7.4)	
**LBBB**	0 (0.0)	0 (0.0)		0 (0.0)	0 (0.0)	

**Table 4b:** Age-related prevalence of chamber and conduction abnormalities between the two groups

ECG abnormality	11 – 13 year-old			14 – 15 year-old		
	VOCN = 15	Steady-state N = 18	p-value	VOC N = 10	Steady-state N = 10	p-value
**LVH**	10 (66.7)	9 (50.0)	0.335^^^	4 (40.0)	4 (40.0)	1.000^^^
**RVH**	1 (6.7)	0 (0.0)	0.204^#^	0 (0.0)	0 (0.0)	-
**BVH**	1 (6.7)	0 (0.0)	0.204^#^	0 (0.0)	0 (0.0)	-
**RAE**	0 (0.0)	0 (0.0)	-	0 (0.0)	0 (0.0)	-
**LAE**	0 (0.0)	1 (5.6)	0.266^#^	0 (0.0)	0 (0.0)	-
**Axis deviation**						
**Absent**	15 (100.0)	16 (88.9)	0.112*	10 (100.0)	9 (90.0)	0.230*
**LAD**	0 (0.0)	2 (11.1)		0 (0.0)	1 (10.0)	
**Heart blocks**						
**Absent**	13 (86.7)	16 (88.9)	0.846*	8 (80.0)	9 (90.0)	0.528*
**First degree**	2 (13.3)	2 (11.1)		2 (20.0)	1 (10.0)	
**Second degree**	0 (0.0)	0 (0.0)		0 (0.0)	0 (0.0)	
**Bundle branch blocks**						
**Absent**	14 (93.3)	16 (88.9)	0.536*	9 (90.0)	10 (100.0)	0.230*
**RBBB**	1 (6.7)	1 (5.6)		1 (10.0)	0 (0.0)	
**LBBB**	0 (0.0)	1 (5.6)		0 (0.0)	0 (0.0)	

Values in parentheses are percentages along columns.

* analysed with Likelihood ratio

# analysed with Yates continuity correction

^ analysed with Pearson chi-squared test.

**BVH:** Biventricular hypertrophy; **LAD:** left axis deviation; **LAE:** left atrial enlargement; **LBBB:** Left bundle branch block; **LVH:** Left ventricular hypertrophy; **RAE:** Right atrial enlargement; **RBBB:** Right bundle branch block; **RVH:** Right ventricular hypertrophy

Combining the different age groups, a higher proportion of the cases, 18 (19.6%), had heart blocks (first and second degree) compared to 7 (7.5%) of the controls (x^2^ = 5.592, p = 0.018). Similarly, the prevalence of prolonged QTc, ST elevation or depression and T wave abnormality was significantly higher in the cases than in the controls. However, the proportion with bundle branch blocks was not significantly different between the cases and the controls (x^2^ = 0.274, p = 0.601). Details of these findings are displayed in Table 5.

**Table 5:** Comparison of the presence of prevalence of heart blocks, ST, and T-wave abnormalities between the two groups

ECG indices	VOC N = 93	Steady-state N = 93	x^2^	P value
**Heart block**				
**Present**	18 (19.4)	7 (7.5)	5.592	**0.018**
**Absent**	75 (80.6)	86 (92.5)		
**Bundle branch block**				
**Present**	9 (9.7)	7 (7.5)	0.274	0.601
**Absent**	84 (90.3)	86 (92.5)		
**Corrected QT interval (QTc)**				
**Not prolonged (≤ 0.44 seconds)**	44 (47.3)	58 (62.4)	4.255	**0.039**
**Prolonged (> 0.44 seconds)**	49 (52.7)	35 (37.6)		
**ST elevation or depression**				
**Absent**	64 (68.8)	76 (81.7)	4.159	**0.041**
**Present**	29 (31.2)	17 (18.3)		
**T wave abnormality**				
**Absent**	64 (68.8)	79 (84.9)	6.806	**0.009**
**Present**	29 (31.2)	14 (15.1)		

## Discussion

The study has demonstrated that some cardiovascular changes accompany vaso-occlusive crisis (VOC) in children with sickle cell anaemia. The mean heart rate in this study was significantly higher in VOC, which agrees with some reports.^12^,^18^ The pain, fever, stress hormones (adrenaline, thyroxine), myocardial ischaemia, and in young children, anxiety that accompanies vaso-occlusive crisis could all work in tandem to increase the heart rate.^27^

This may be a compensatory mechanism to increase oxygen supply to important tissues because of the ischaemia that occurs during VOC. Previous reports have documented lower blood pressures in patients with SCA compared to non-SCA patients.^17^,^19^,^28^

Factors responsible for this include renal salt wasting, reduced sensitivity to nitric oxide and autonomic dysfunction.^17^,^29^,^30^ However, during VOC, the blood pressure of SCA patients may increase. Elevated blood pressure during VOC increases the risk of stroke, myocardial ischaemia and pulmonary hypertension, although this has not been widely reported in children.^19^,^31^ Although there was no significant difference in the blood pressure distribution between the steady state children and those with VOC in this study, four children with VOC had elevated blood pressure as against none in the control group. A previous report showed an increased risk for stroke with systolic but not diastolic hypertension.^31^

This gives further clinical relevance to this study's significantly higher mean systolic, diastolic and mean arterial pressures in VOC. None of the participants in this study had a stroke. However, it is important to monitor the blood pressure in the routine care of children with SCA and, more importantly, during VOC to prevent these acute sinister complications.

There were no statistically significant differences in the mean ECG indices assessed in the participants (PR interval, P wave duration, QT interval, QTc interval and P, QRS and T axes). Notwithstanding, the higher mean QTc in VOC in this study is of clinical significance, and similar findings were recently reported by Anah *et al.*^32^ Prolonged QTc interval had been reported in children with SCA both in steady state and during VOC when compared with haemoglobin AA.^12^,^33^ This prolongation is associated with a higher risk of cardiac arrhythmias and sudden death.^13^,^34^ The higher mean QTc value in this study suggests a further increased risk during VOC, which clinicians must be aware of and should guide therapy, especially with medications that could further prolong the QTc interval.

Similar to previous reports in children and adults with SCA,^12^,^15^,^33^ left ventricular hypertrophy (LVH) was the most common chamber enlargement identified on ECG in this study. No significant difference between the two groups was found in the prevalence of LVH and other chamber enlargements. This may reflect the chronic nature of these changes, which occur over time both in crisis and in steady-state as compensation for the chronic haemolytic state and increased wall stress in SCA, culminating in hyperdynamic and restrictive physiology.^8^,^10^,^16^,^35^ Wide variations exist in the prevalence of chamber hypertrophy from different studies, possibly due to variations in other clinical and laboratory parameters that have been shown to influence these changes (steady state haematocrit, sleep and awake oxygen concentrations, use of hydroxyurea.)^36^–^38^ which were not all assessed or reported by the various studies.

Abnormalities in the cardiac conduction system were significantly present during VOC than in steady- state in this study. Similarly, ECG features suggestive of myocardial ischaemia were significantly more prevalent during VOC. These include ST elevations and depression, and T-wave changes. Subclinical ischaemia has been shown to occur in SCA even in steady state,^33^,^39^-^40^, and this is thought to result from factors that include abnormal vasculature, vasospasm and increased adherence of sickled red blood cells to the vascular endothelium.^35^ These factors likely worsen during VOC, culminating in increased ischaemia.

Findings from studies by Adeniyi *et al.*^41^ and Nlemadim *et al.*^20^ showed that evidence of myocardial ischaemia is significantly higher during VOC than in-steady state. Wagdy *et al.* also reported biochemical evidence of myocardial ischaemia (cardiac troponin I) and fibrosis (galectin-3) in patients with VOC.^40^ This worse ischaemia during VOC could affect the myocardial conducting system and present as heart and bundle branch blocks, as reflected in the higher though not significant presence of bundle branch blocks and significantly higher prevalence of heart blocks during VOC in this study. These factors could further contribute to mortality in SCA patients with VOC.

The study has some limitations. Echocardiography and cardiac enzymes would have given more information about the cardiac function in both study groups. A follow-up assessment of the cases after recovery from the acute VOC could also have further demonstrated the link between the crisis state and cardiovascular changes. However, the study's control group with similar baseline characteristics has partly made up for this.

## Conclusion

This study has shown that VOC in SCA are characterised by deeper pathophysiologic changes in cardiovascular function and structure beyond the acute painful episodes. Although cardiovascular abnormalities like hypotension or hypertension, ECG features of myocardial ischaemia (prolonged QTc, ST-T changes) and cardiac conduction abnormalities (heart blocks) are seen in SCA even in steady-state, they are worse during VOC, and these might increase the risk of arrhythmias and sudden death. We advocate that physicians be wary of these changes and be on the lookout for them to reduce the risk of mortality during VOC.

## References

[1] CDC (2021). Data & Statistics on Sickle Cell Disease ∣ CDC [Internet].

[2] National Population Commission N (2020). Nigerian Demographic and Health Survey 2018 [Internet].

[3] Lanzkron S, Carroll CP, Haywood C (2013). Mortality rates and age at death from sickle cell disease: US, 1979-2005. Public Health Rep Wash DC.

[4] Nnodu OE, Oron AP, Sopekan A, Akaba GO, Piel FB, Chao DL (2021). Child mortality from sickle cell disease in Nigeria: a model-estimated, population-level analysis of data from the 2018 Demographic and Health Survey. Lancet Haematol.

[5] Odame I, Jain D (2020). Sickle cell disease: Progress made & challenges ahead. Indian J Med Res.

[6] Odièvre M-H, Verger E, Silva-Pinto AC, Elion J (2011). Pathophysiological insights in sickle cell disease. Indian J Med Res.

[7] Rees DC, Williams TN, Gladwin MT (2010). Sickle-cell disease. The Lancet.

[8] Rai P, Niss O, Malik P (2017). A reappraisal of the mechanisms underlying the cardiac complications of sickle cell anemia. Pediatr Blood Cancer.

[9] Bakeer N, James J, Roy S, Wansapura J, Shanmukhappa SK, Lorenz JN (2016). Sickle cell anemia mice develop a unique cardiomyopathy with restrictive physiology. Proc Natl Acad Sci.

[10] Niss O, Quinn CT, Lane A, Daily J, Khoury PR, Bakeer N (2016). Cardiomyopathy With Restrictive Physiology in Sickle Cell Disease. JACC Cardiovasc Imaging.

[11] Antwi-Boasiako C, Dankwah GB, Aryee R, Hayfron-Benjamin C, Donkor ES, Campbell AD (2019). Oxidative Profile of Patients with Sickle Cell Disease. Med Sci Basel Switz.

[12] Adegoke SA, Okeniyi JA, Akintunde AA (2016). Electrocardiographic abnormalities and dyslipidaemic syndrome in children with sickle cell anaemia. Cardiovasc J Afr.

[13] Indik JH, Nair V, Rafikov R, Nyotowidjojo IS, Bisla J, Kansal M (2016). Associations of Prolonged QTc in Sickle Cell Disease. PLoS ONE.

[14] Oguanobi NI, Onwubere BJC, Ike SO, Anisiuba BC, Ejim EC, Ibegbulam OG (2010). Electocardiographic findings in adult Nigerians with sickle cell anaemia. Afr Health Sci.

[15] Dosunmu A, Akinbami A, Uche E, Adediran A, John-Olabode S (2016). Electrocardiographic Study in Adult Homozygous Sickle Cell Disease Patients in Lagos, Nigeria. J Trop Med. [Internet].

[16] Gladwin MT (2017). Cardiovascular complications in patients with sickle cell disease. Hematol Am Soc Hematol Educ Program.

[17] Hussain A, Hassan M (2021). Blood Pressure of Children and Adolescents with Sickle Cell Anemia in Basra, Iraq. undefined [Internet].

[18] Onalo R, Cooper P, Cilliers A, Nnebe-Agumadu U (2020). Cardiovascular changes in children with sickle cell crisis. Cardiol Young.

[19] Onalo R, Cilliers A, Cooper P (2021). Impact of oral L-arginine supplementation on blood pressure dynamics in children with severe sickle cell vaso-occlusive crisis. Am J Cardiovasc Dis.

[20] Nlemadim AC, Okpara HC, Anah MU, Odey FA, Meremikwu MM (2020). Myocardial injury in patients with sickle cell anaemia and myocardial ischaemia in Calabar, Nigeria. Paediatr Int Child Health.

[21] Borhade MB, Kondamudi NP (2021). Sickle Cell Crisis. StatPearls [Internet].

[22] Kasiulevicius IV, Sapoka IV, Filipaviciute R (2006). Sample size calculation in epidemiological studies. Gerontologic.

[23] Bode-Thomas F, Hyacinth HI, Ogunkunle O, Omotoso A (2011). Myocardial ischaemia in sickle cell anaemia: evaluation using a new scoring system. Ann Trop Paediatr.

[24] Ballas SK, Lieff S, Benjamin LJ, Dampier CD, Heeney MM, Hoppe C (2010). Definitions of the Phenotypic Manifestations of Sickle Cell Disease. Am J Hematol.

[25] Kolawole AJ, Omokhodion SI (2014). Normal limits for pediatric electrocardiogram in Ilorin, Nigeria. Nig J Cardiol.

[26] National High Blood Pressure Education Program Working Group on High Blood Pressure in Children and Adolescents (2004). Pediatrics.

[27] Tennant F (2013). The physiologic effects of pain on the endocrine system. Pain Ther.

[28] Ernst AA, Weiss SJ, Johnson WD, Takakuwa KM (2000). Blood pressure in acute vaso-occlusive crises of sickle cell disease. South Med J.

[29] Nath KA, Hebbel RP (2015). Sickle cell disease: renal manifestations and mechanisms. Nat Rev Nephrol.

[30] Martins W de A, Lopes HF, Consolim-Colombo FM, Gualandro S de FM, Arteaga-Fernández E, Mady C (2012). Cardiovascular autonomic dysfunction in sickle cell anemia. Auton Neurosci.

[31] Pegelow CH, Colangelo L, Steinberg M, Wright EC, Smith J, Phillips G (1997). Natural history of blood pressure in sickle cell disease: risks for stroke and death associated with relative hypertension in sickle cell anemia. Am J Med.

[32] Anah M, Nlemadim A, Uzomba C, Ineji EO, Odey F (2021). Prolonged QTc Interval in Nigerian Children with Sickle Cell Anemia. Hemoglobin.

[33] Odia K, Dapper V, George I, Olorunfemi OJ (2016). Changes in some electrocardiographic parameters amongst children with sickle cell anemia in Port Harcourt, Nigeria. IOSR-JDMS.

[34] Upadhya B, Ntim W, Brandon Stacey R, Henderson R, Leedy D, O'Brien FX (2013). Prolongation of QTc intervals and risk of death among patients with sickle cell disease. Eur J Haematol.

[35] Voskaridou E, Christoulas D, Terpos E (2012). Sickle-cell disease and the heart: review of the current literature. Br J Haematol.

[36] Ikegwuonu C, Ezeanosike O, Manyike C, Ujunwa F, Ikegwuonu C (2021). Ventricular hypertrophy and ischaemic changes in Children with Sickle Cell Anaemia and its relationship with the Haemoglobin Concentration in Steady State. Med Sci Discov.

[37] Johnson MC, Kirkham FJ, Redline S, Rosen CL, Yan Y, Roberts I (2010). Left ventricular hypertrophy and diastolic dysfunction in children with sickle cell disease are related to asleep and waking oxygen desaturation. Blood.

[38] Faro GB de A, Menezes-Neto OA, Batista GS, Silva-Neto AP, Cipolotti R (2015). Left ventricular hypertrophy in children, adolescents and young adults with sickle cell anemia. Rev Bras Hematol E Hemoter.

[39] Akinola NO, Stevens SM, Franklin IM, Nash GB, Stuart J (1992). Subclinical ischaemic episodes during the steady state of sickle cell anaemia. J Clin Pathol.

[40] Wagdy R, Suliman H, Bamashmose B, Aidaroos A, Haneef Z, Samonti A (2018). Subclinical myocardial injury during vaso-occlusive crisis in pediatric sickle cell disease. Eur J Pediatr.

[41] Adeniyi AT, Okeniyi JAO, Adegoke SA, Oseni SBA, Smith OS, Abe-Dada AA (2021). Clinical Utilities of Electrocardiography in the Diagnosis of Myocardial Ischemia in Children With Sickle Cell Anemia: Correlation With Serum Cardiac Troponin I. J Pediatr Hematol Oncol.

